# Measuring CO_2_ Concentration and Thermal Comfort in Italian University Classrooms: A Seasonal Analysis

**DOI:** 10.3390/s25071970

**Published:** 2025-03-21

**Authors:** Alessia Fedele, Andrea Colantoni, Giuseppe Calabrò, Mauro Scungio, Stefano Rossi, Juri Taborri

**Affiliations:** 1 Department of Economics, Engineering, Society and Business Organization, University of Tuscia, 01100 Viterbo, Italy; alessiafedele96@gmail.com (A.F.); giuseppe.calabro@unitus.it (G.C.); mauro.scungio@unitus.it (M.S.); stefano.rossi@unitus.it (S.R.); 2 Department of Agriculture and Forest Sciences, University of Tuscia, 01100 Viterbo, Italy; colantoni@unitus.it

**Keywords:** thermal comfort, CO_2_ concentration, sensor systems

## Abstract

This study investigates indoor air quality (IAQ) and thermal comfort in Italian university classrooms, considering seasonal variations. Poor IAQ can impair students’ cognitive performance and well-being, making ventilation strategies crucial. The aim is to assess CO_2_ levels, temperature, and humidity, analyzing their interactions across seasons. A monitoring protocol was applied in three classrooms using NDIR sensors and a microclimate assessment system. Sensors were placed strategically to capture representative data in 20 days in spring and autumn. Results indicate that CO_2_ levels peaked at 2324.2 ppm in autumn, significantly exceeding the 1000 ppm threshold, whereas spring levels remained below 953.4 ppm. Relative humidity ranged from 32.7% to 55.6%, with higher values in autumn. Temperatures varied from 19.1 °C to 27.5 °C, with warmer conditions in spring. Strong positive correlations (always greater than 0.70) between CO_2_ and humidity suggest inadequate air exchange reduces IAQ, potentially affecting cognitive performance. This research provides valuable insights for improving student well-being through better air quality management. This research provides valuable insights for optimizing classroom environments, supporting cognitive performance, and improving student well-being through better air quality management.

## 1. Introduction

Indoor air quality (IAQ), mainly CO_2_ concentration, and thermal comfort are increasingly recognized as critical elements influencing health and well-being [1,2]. IAQ and thermal comfort are also demonstrated to be essential components to guarantee a healthy learning environment, reducing absence rate and improving test scores, as well as cognitive performance, in educational settings [3,4]. Classrooms are often subject to fluctuating and elevated levels of carbon dioxide (CO_2_) due to high occupancy, limited space, and varying ventilation quality, which can lead to suboptimal conditions for learning [5,6]. Research underscores the significance of maintaining CO_2_ concentrations below 1000 parts per million (ppm), as higher levels have been shown to impair cognitive abilities essential for academic tasks [7]. For instance, students exposed to environments with high CO_2_ concentrations display reduced problem-solving skills, memory retention, and information processing, which collectively diminish academic performance [8].

Studies on indoor air quality in educational environments indicate that CO_2_ concentration serves as a reliable marker for assessing ventilation adequacy and occupancy levels. For example, Hui et al. found that indoor CO_2_ levels could serve as a proxy for ventilation efficiency, as higher concentrations indicate insufficient fresh air exchange [9]. As a direct product of human respiration, CO_2_ accumulates rapidly in enclosed spaces with inadequate air exchange, creating environments with lower oxygen availability and higher levels of contaminants. Several investigations into the CO_2_ concentration of schools and universities show that the concentration of CO_2_ tends to rise considerably during class hours, peaking at levels that can exceed recommended standards, particularly in colder seasons when natural ventilation (e.g., open windows) is minimized to maintain thermal comfort [10]. Previous studies have consistently highlighted the seasonal impact on CO_2_ concentration in classrooms, with higher levels typically recorded during the winter months due to reduced ventilation practices. Investigations conducted in various educational settings, including naturally ventilated classrooms in different climates, have shown that CO_2_ accumulation is significantly influenced by outdoor temperatures, as lower temperatures discourage window opening and natural ventilation [11,12]. In particular, a comparison study between Polish and Spanish classrooms reported consistently higher CO_2_ levels in the coolest region, i.e., Poland, with a pronounced effect in the early morning hours, suggesting a strong correlation between seasonal ventilation habits and indoor air quality [12]. A similar trend has been observed in Italian primary schools, where research has demonstrated that manual airing techniques alone are not sufficient to maintain acceptable indoor air quality during the heating season [13]. Furthermore, the study emphasized that while spring conditions may allow for more effective natural ventilation, wintertime air exchange remains inadequate, requiring alternative solutions to maintain CO_2_ concentrations within recommended thresholds. In addition to CO_2_ accumulation, research also suggests that high levels of indoor pollutants, such as particulate matter (PM), can coincide with increased CO_2_ concentrations, particularly in schools where manual airing strategies are employed inconsistently [14].

In addition to IAQ, there has been a growing interest in research analyzing the role of thermal comfort parameters—namely temperature and relative humidity—on both physiological and cognitive functions within classroom environments [15,16]. Temperature fluctuations, even within mild ranges, can influence students’ heart rates and thermal perceptions, potentially inducing discomfort that affects their focus and task performance [17]. Studies by Brink et al. indicate that both high and low extremes of thermal conditions disrupt cognitive tasks such as memory recall and vigilance [18], even if it is worth underlining that, regarding the thermal perception, female students tend to feel colder than their male counterparts. Similarly, Barbic et al. observed a decrease of up to 24% in cognitive performance among students experiencing thermal discomfort due to high temperature [19]. However, not all thermal discomfort sensations lead to a deterioration in cognitive performance, and the impact is most likely activity-dependent; in fact, a cooler environment has been demonstrated to positively influence cognitive performance in comparison to the colder and warmer ones. Moreover, the thermal sensation ’warm’ influences cognitive performance in tasks related to vigilance, memory and learning more than the thermal sensation ‘cold’. Evidence suggests a consistent relationship between CO_2_ levels and other microclimate variables. Specifically, research by Lazović et al. highlighted that the correlation between CO_2_ concentration and relative humidity can serve as a key indicator of air quality and ventilation efficiency, especially in naturally ventilated spaces [20]. For example, high occupancy without adequate air exchange often results in both increased CO_2_ levels and higher relative humidity due to human exhalation and respiration processes [21]. These interconnected variables suggest that managing one factor can often influence the others, underscoring the importance of a comprehensive IAQ-monitoring approach that encompasses CO_2_, temperature, and humidity [22].

From this perspective, it is clear how a sensor-based measurement of not only CO_2_ levels but also temperature and humidity is mandatory in classrooms to sustain a productive learning atmosphere and to properly design and manage classroom ventilation strategies, moreso than in other types of buildings, such as the ones dedicated for office work [17,23]. Even if a plethora of studies have been already proposed in the literature, to the best of the authors’ knowledge, analyses are limited to specific seasons and specific geographical areas. In addition, university classrooms, characterized by high occupancy levels and variable ventilation conditions, are particularly susceptible to fluctuations in CO_2_ concentration, temperature, and humidity. While extensive research has been conducted on indoor air quality in primary and secondary schools, fewer studies have focused on higher education environments, where students typically spend extended periods in enclosed spaces with limited ventilation control. In light of these findings, the present study investigates the seasonal variations in CO_2_ concentration and thermal comfort in university classrooms, aiming to assess how different ventilation practices impact air quality throughout the academic year, especially in the seasons with higher occupancy due to lectures rather than exams, which are spring and autumn. Our central hypothesis is that CO_2_ levels significantly increase during autumn due to reduced natural ventilation, leading to higher relative humidity and potential discomfort for students, whereas spring conditions allow for improved air exchange, maintaining better indoor air quality. To test this hypothesis, we conducted continuous environmental monitoring across different classroom settings, analyzing the relationship between CO_2_ concentration, humidity, and thermal comfort. By focusing on university classrooms, this study addresses a critical gap in the literature and provides practical insights for optimizing ventilation strategies in higher education settings. These findings contribute to a growing body of research on indoor air quality management and offer evidence-based recommendations for improving student well-being and learning conditions in academic institutions.

## 2. Materials and Methods

### 2.1. Experimental Setup

**Figure 1:**
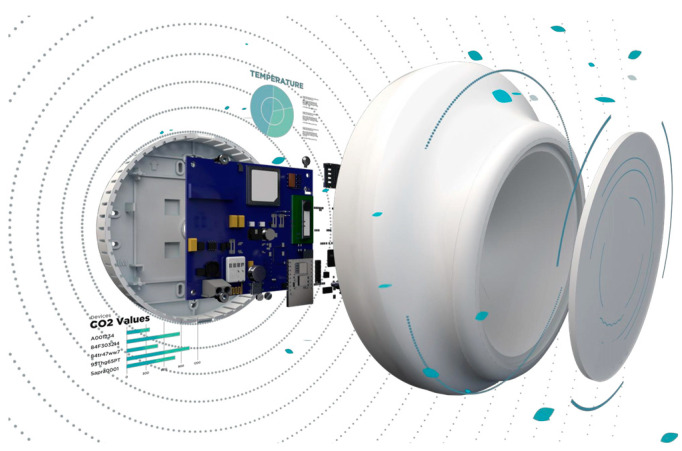
Structure of the NDIR sensor.

For the objective of measuring carbon dioxide concentration, temperature and humidity, a commercially available Non-Dispersive Infrared (NDIR) sensor produced by Fybra (model: Fybra School, Milan, Italy) has been used (Figure 1).

The working principle of the sensor for the measurement of the carbon dioxide concentration is based on spectroscopy, specifically on the energy absorption characteristics of CO_2_ in the infrared zone. The NDIR sensor has an infrared light source that emits through a measuring chamber. The gas to be measured flows within this chamber. The infrared light traverses the gas sample, and the gas molecules absorb energy at specific infrared wavelengths. Each gas possesses its own unique infrared absorption spectrum. For instance, in the case of CO_2_, absorptions of radiation with wavelengths at 2.7 µm, 4.7 µm and 15 µm are detected in the infrared region. Following the passage of infrared light through the gas sample, it reaches a detector responsible for measuring the intensity of the light striking it. The difference between the intensity of the emitted infrared light and the intensity of the detected light offers insights into the gas absorption within the sample. The same sensor is also able to gather measurements on temperature (T) and relative humidity (RH). Metrological characteristics of Fybra sensors are reported in Table 1.

**Figure 2:**
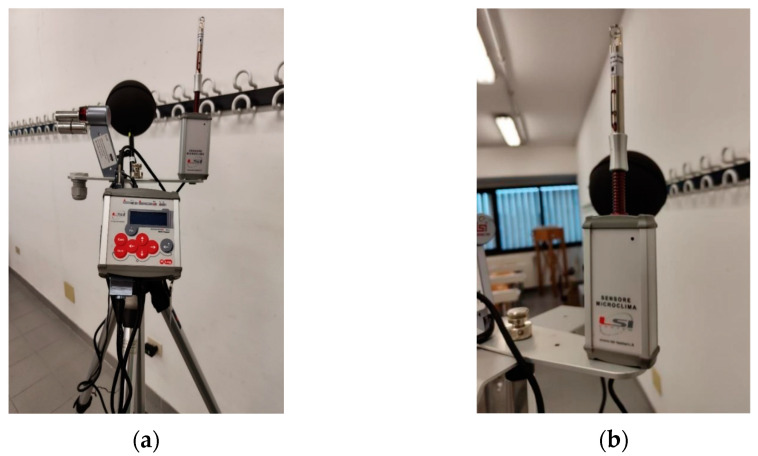

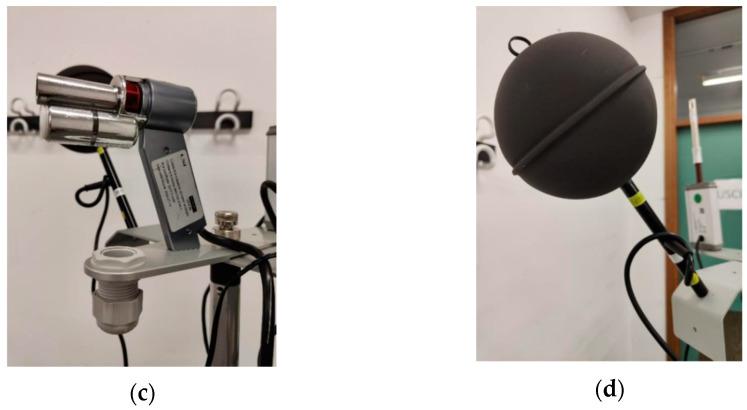
(**a**) overall BABUC system for microclimate assessment; (**b**) hot-wire anemometer; (**c**) psychrometer; and (**d**) globe thermometer.

For the microclimate assessment, the BABUC (LSI Lastem srl, Milan, Italy) system has been adopted. The system consists of an instrument assembly (data logger and sensors) mounted on a tripod, as depicted in Figure 2. Depending on the specific environment and the related microclimatic survey (moderate, hot and cold environments and localized discomforts), it is possible to choose different types of sensors. For our application, a moderate indoor environment was analyzed, and therefore the utilized probes consisted of a *hot*-*wire anemometer* (model ESV126, LSI Lastem srl, Milan, Italy), a *psychrometer* (model MINI-DIN ESU102A, LSI Lastem srl, Milan, Italy) and a *globe thermometer* (model DMA131A, LSI Lastem srl, Milan, Italy).

Through the sensor system, we gathered the following measures: (i) flow velocity; (ii) dry air temperature; (iii) wet air temperature; (iv) relative humidity; and (v) mean radiant temperature. The BABUC system has been already used in the literature for microclimatic analysis in indoor environments [24,25]. Metrological characteristics of the BABUC system are reported in Table 2.

### 2.2. Experimental Protocol

The monitoring and evaluation of CO_2_ concentration and thermal comfort was conducted in three classrooms of varying size at the University of Tuscia, located in the School of Engineering. The selected classrooms included the following: (i) F8-97 m^2^, volume 291 m^3^, maximum capacity of 70 students; (ii) F9-78.23 m^2^, volume 234.69 m^3^, maximum capacity of 54 students; and (iii) B1-112.40 m^2^, volume 337.20 m^3^, maximum capacity of 40 students.

Each classroom is characterized by the following:

- Ventilation system—Natural ventilation through windows;
- Windows—B1 and F8 are equipped with 4 single-glazed aluminum inward-opening windows; whereas F9 with 2 single-glazed aluminum inward-opening windows.
- Equipment—Desks, chairs, projector, whiteboard;
- Heating system—Central heating with radiators along walls;
- Location and orientation: low-traffic area surrounded by open fields and east-to-northeast orientation; the direct sunlight exposure is primarily limited to the morning hours.

Given the sizes of the classrooms, sensor placement was optimized to capture representative data for each environment. Classrooms F8 and F9 were equipped with one sensor each, positioned at the front of the room near the desk and front rows. In Classroom B1, due to its larger size, two sensors were installed: one near the desk and front rows, and another at the rear of the room. The placement of sensors within the classrooms adhered strictly to the following criteria, as recommended by ISO 16000:26 and GdS Indoor Pollution guidelines:

- *Height*: Sensors were installed at approximately 1.5 m above the floor, which corresponds to the breathing zone of seated occupants. This placement minimized measurement bias from non-representative air strata.
- *Distance from Occupants*: Sensors were placed 1.5–2 m away from occupants to reduce direct interference from exhaled air.
- *Distance from Obstacles*: A minimum distance of 1 m was maintained from walls, bookshelves, and other potential obstructions to ensure an unobstructed measurement of the indoor air.
- *Ventilation Interference*: Sensors were positioned away from direct airflow caused by windows, doors, or air conditioning units to avoid artificial fluctuations in readings.
- *Heat Sources*: Care was taken to avoid proximity to radiators, spotlights, or other heat sources that could influence temperature and relative humidity measurements.

In addition to continuous monitoring, the sensors were equipped with a visual alert system to provide real-time feedback to occupants: (i) blue, indicating good air quality; (ii) red, indicating poor air quality, prompting immediate ventilation; (iii) and violet, denoting improving air quality. Specifically, the red light starts when CO_2_ overcomes the limit of 1000 ppm, whereas the violet starts at 900 ppm. Values lower than 900 are associated with blue light.

Data acquisition through NDIR sensors was performed in two distinct periods, spring and autumn, to capture seasonal variations in indoor air quality and thermal comfort conditions. The data collection schedule comprised 50 days between May 2022 and December 2022, twenty-five per each season, for the three above-mentioned classrooms. Details of students’ occupancy are reported in Table 3.

In all classes of the case study, the lecture starts at 9:00 a.m. Depending on the type of activities, they last 60 or 120 min, and after that a 15 min break takes place. A long break is scheduled from 1 to 2 pm. During both autumn and spring, windows were kept closed before the school start time, whereas the classroom’s door was kept open till the lesson start. During the lecture time, a different behavior was recognized in autumn and spring: (i) in the autumn, windows and doors were kept ordinarily closed (aside from the previously mentioned break time, during which both windows and doors were opened to let fresh air enter the classroom); (ii) in spring, windows are mainly kept open, whereas the door was kept closed (expect during the break time). The specifications of this case study are in line with other previously available case studies in the literature [11,12,13,26].

These timeframes were chosen to reflect different external environmental conditions, influencing natural ventilation behavior, and occupancy patterns. Outputs of the BABUC system were collected for a complete microclimate assessment only in classroom F8 due to its higher student occupancy, making it a critical space for IAQ evaluation.

On each survey day, sensors were calibrated and the actual data acquisition started after 30 min to guarantee the stabilization of the signals. Measurements were recorded every 120 s to ensure consistent temporal resolution across all monitored variables. This interval was selected to align with the response times of the sensors. The measurement duration for each classroom spanned the full teaching schedule, capturing occupancy dynamics and ventilation practices throughout the day.

For the microclimate analysis, subjective thermal comfort was assessed through questionnaires distributed to students present during the survey to assess the Predicted Mean Vote (PMV). Participants rated their thermal sensation on a 7-point scale ranging from −3 (very cold) to +3 (very warm), as in the following Table 4, as requested in the norm ISO 7730:2006:

### 2.3. Data Analysis

The collected data were exported in CSV format, with separate files created for each variable and classroom. The data analysis was conducted using MATLAB software (version R2023a) to process, evaluate, and interpret the datasets collected from the NDIR sensors and the BABUC microclimate station.

Concerning NDIR sensors, data were firstly structured into matrices, categorized by four daily intervals, which were as follows: (i) 9–11 a.m.; (ii) 11 a.m.–1 p.m.; (iii) 2–4 p.m.; and (iv) 4–6 p.m.. It is worth noticing that the interval 1–2 p.m. has been excluded by the successive analysis for the absence of occupants in the classroom due to the lunch break. Successively, descriptive statistics, including maximum, minimum, mean, and standard deviation, were computed for CO_2_ concentration, temperature and relative humidity per each daily interval. It is worth highlighting that the results for the classroom B1, in which two NDIR sensors were placed, were obtained by firstly averaging the measurements of the two sensors. Apposite graphs were realized to monitor when CO_2_ concentration overcame the norm threshold of 1000 ppm.

Moving to the microclimate analysis through the BABUC system, performed only in classroom F8, we applied the approach based on ISO 7730:2006, which defines thermal comfort using the Predicted Mean Vote (PMV) and Predicted Percentage of Dissatisfied (PPD) indices. Using the replies to the questionnaire, the PPD index was calculated by using the following formula:

$$PPD\;=\;100\;-\;95e^{\left(-0.03353{PMV}^{4}-0.2179{PMV}^{2}\right)}$$

The previous equation can be applied only if the following values are met: (i) indoor air temperature in the range 10–30 °C; (ii) average radiant temperature in the range 10–40 °C; and (iii) air velocity in the range 0–1 m/s. The coherence with the requirement has been verified by using data gathered from the BABUC system.

### 2.4. Statistical Analysis

Correlation between CO_2_ concentration and relative humidity was assessed. Scatter plots were created with relative humidity values on the x-axis and CO_2_ concentrations on the y-axis, and a trend line was fitted to visualize the relationship. Pearson’s correlation coefficient (r) was calculated to quantify the strength of the relationship. Correlation coefficients approaching +1 indicated a strong positive correlation, values near −1 indicated a strong negative correlation, and values close to 0 suggested no significant relationship. Furthermore, to determine whether significant differences in mean CO_2_ concentrations existed across different time intervals, a one-way ANOVA test was conducted. Bonferroni’s multiple comparison test was performed in case of significant ANOVA results. Seasonal differences in CO_2_ concentrations and PMV between spring and autumn were further examined using a two-sample *t*-test, independently per each variable.

## 3. Results and Discussions

### 3.1. Temperature, Relative Humidity and CO_2_ Concentration: Seasoning Variations and Correlation

**Figure 3:**
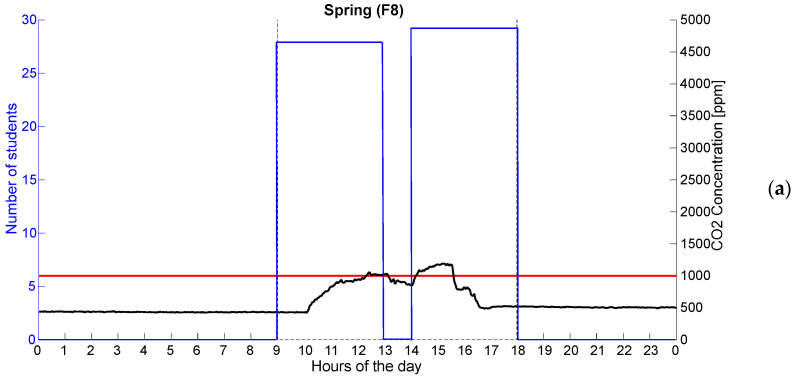

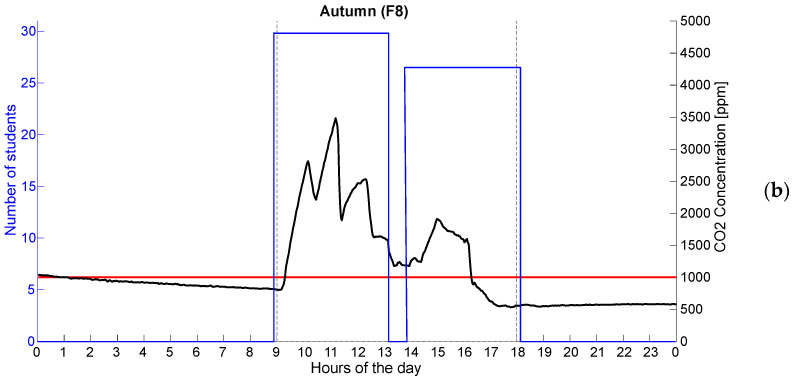
Example of the trend of CO_2_ concentration in F8 during spring (**a**) and autumn (**b**). Solid red, black and blue lines represent the CO_2_ threshold, the trend of CO_2_ and the number of occupants, respectively. Dotted black line indicates the tested time intervals.

Figure 3a,b reports an example of the behavior of the CO_2_ concentration in one classroom in relation to the time intervals and the number of occupants. Qualitatively, the graphs compare CO_2_ concentration and student presence in a classroom during spring and autumn. In both cases, CO_2_ levels increase when students are present and decrease after they leave, highlighting the direct relationship between occupancy and indoor air quality. Anyway, even if it is true that when windows and doors were opened the CO_2_ concentration fell (in the break 1–2 pm), the concentration suddenly started increasing again when the openings were fully closed. Thus, the short-term manual airing periods were insufficient to maintain average CO_2_ concentrations below 1000 ppm, especially in autumn. This qualitative example is in line with the results reported in [13], also considering similar climate regions.

The red line represents a threshold beyond which CO_2_ levels may become critical, and in autumn, the concentration exceeds this limit more significantly than in spring.

Table 5 summarizes the mean, maximum, and minimum values (with standard deviations) for T, RH, and CO_2_ concentration in three rooms (F8, F9, and B1) across two seasons (spring and autumn), considering whole-day measurements. 

During spring, the mean temperature ranged from 23.5 °C in F8 to 24.5 °C in B1, with maximum values reaching 27.5 °C in F9 and minimum values as low as 22.4 °C in F9. RH was generally lower in spring, with mean values between 32.7% (F9) and 36.5% (F8). Maximum RH peaked at 39.4% in F8, while minimum RH values went as low as 27.4% in F9. CO_2_ levels showed substantial variation, with mean concentrations highest in B1 (547.4 ppm) and lowest in F8 (492.0 ppm). Maximum CO_2_ levels peaked at 953.4 ppm in F9, whereas minimum values were consistent across rooms, with F9 recording the lowest minimum at 419.2 ppm. In autumn, temperatures were slightly lower, with mean values ranging from 21.0 °C in F8 to 22.9 °C in B1. Maximum temperatures reached 24.5 °C in B1, while minimum temperatures dropped to 19.1 °C in F8. Relative humidity was notably higher in autumn compared to spring, with mean values ranging between 49.4% (B1) and 51.6% (F8). Maximum RH reached 55.6% in F8, while minimum values were lowest at 46.0% in B1. CO_2_ concentrations exhibited a pronounced seasonal increase in autumn, particularly in F8, where the mean reached 1034.0 ppm with a maximum of 2324.2 ppm. In contrast, minimum CO_2_ levels across rooms remained comparable, with values between 457.0 ppm (B1) and 475.8 ppm (F9). Statistical differences between the two seasons were found for each mean parameter and for all the rooms, with *p*-values ranging from <0.01 and 0.04. Moving to the time interval analysis, no statistical differences were found, with all the ANOVA tests associated with *p*-values ranging from 0.10 to 0.85.

Overall, the results highlight seasonal variations, with higher CO_2_ concentrations and relative humidity observed in autumn and higher temperatures recorded during spring. The observed increase in RH and CO_2_ concentrations during the autumn season, compared to spring, can be attributed to several environmental and seasonal factors, as the effects induced by occupancy can be considered negligible since, during the survey period, the occupancy of each room remained similar, ranging consistently between 25 to 30 students. This stable occupancy ensures that variations in CO_2_ and RH levels are not influenced by differences in the number of occupants but rather by other external or environmental factors. Similar results have been reported in [11], where an increment of both CO_2_ concentration and RH has been found in the winter semester in comparison to the summer one when seeking to evaluate the indoor environment quality in Slovakian classrooms. The results are also consistent in terms of students’ occupancy that is comparable, as well as the use of only natural ventilation. Likewise, when compared different climates (Poland vs. Spain), the coolest region was found to be related to higher concentrations of carbon dioxide, revealing the influence of external temperature [12]. However, it is worth noting that in the reported study, the differences are significant, especially during the first hours of the day, whereas in our study no differences among hours were found. The increment in CO_2_ concentration is also in line with the results proposed in [13], where the analysis of indoor air quality has been conducted in primary school classrooms with natural ventilation and manual airing, as in this study. Data clearly highlight that students were exposed to high CO_2_ concentrations in the cooler season also in [13,14,26].

Focusing on the reasons, the rise in CO_2_ levels during autumn may be explained primarily by changes in ventilation practices, confirming the results presented in [5,10]. Cooler outdoor temperatures in autumn often result in reduced natural ventilation as windows and doors are kept closed to maintain indoor thermal comfort. Limited ventilation diminishes the air exchange rate, causing CO_2_ exhaled by occupants to accumulate indoors. Studies have shown that ventilation rates significantly influence indoor CO_2_ concentrations, with insufficient ventilation resulting in higher indoor CO_2_ levels, especially in occupied spaces. RH is also elevated during autumn due to a combination of seasonal and indoor factors. Cooler temperatures reduce the air’s capacity to hold moisture, causing a higher relative humidity for the same amount of water vapor present [27]. Additionally, reduced ventilation practices may trap moisture indoors, further contributing to elevated RH levels. Indoor sources of humidity, such as human respiration, can exacerbate the rise in RH in enclosed spaces. This phenomenon is supported by research highlighting the importance of ventilation in controlling indoor humidity, particularly during cooler seasons when natural ventilation is restricted [28]. Seasonal climatic differences further compound these indoor changes. In many climates, autumn is characterized by increased precipitation and cooler weather, which contribute to higher outdoor humidity levels [29]. This outdoor air, when introduced indoors via mechanical ventilation or infiltration, can raise indoor RH levels. Furthermore, it is worth highlighting that, as previously reported, the buildings have an east-to-northeast orientation, meaning that direct sunlight exposure is primarily limited to the morning hours. This orientation influences indoor thermal conditions, as classrooms receive solar radiation in the early hours but remain largely shaded in the afternoon. Consequently, natural heating effects from solar exposure are reduced later in the day, potentially affecting indoor temperature and humidity dynamics, particularly during colder months when ventilation practices are more restricted.

In summary, it is evident that relying solely on manual airing based on subjective physical perception or during the break is not sufficient to ensure minimum indoor air quality in university environments, especially during autumn when severe meteoclimatic conditions can worsen indoor air stagnation. However, this method might still be effective in milder conditions. Based on the findings, even in cases where mechanical ventilation systems cannot be implemented due to cost and structural constraints, it is crucial to adopt alternative solutions that facilitate effective ventilation during critical indoor air quality situations. The simplest and most cost-effective approach could involve establishing detailed window-opening protocols.

**Figure 4:**
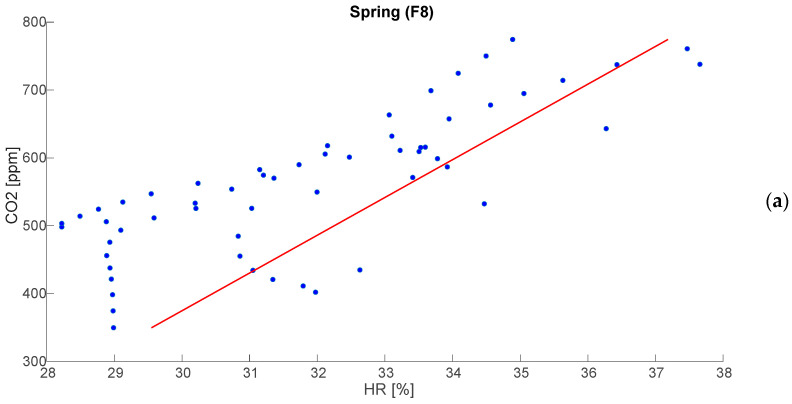

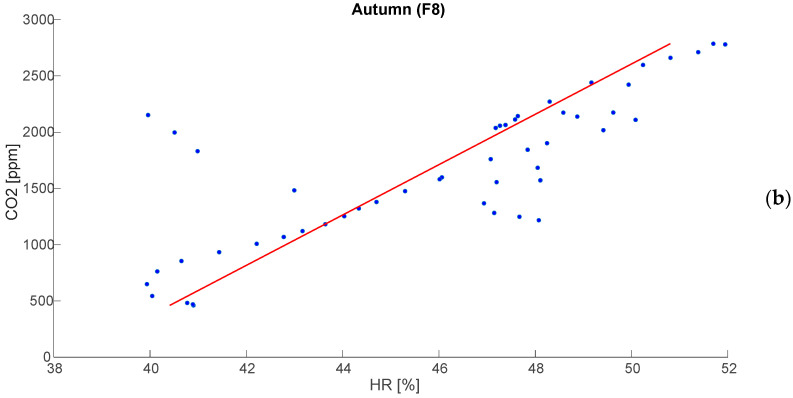
Example of correlation between CO_2_ concentration and RH in F8 during spring (**a**) and autumn (**b**). Blue points represent the data, whereas the solid red line is the regression line.

Figure 4a,b report an example of correlation betwenn CO_2_ and RH for classroom F8 in both tested seasons. Table 6 reports all the r values across seasons and classrooms.

The results indicate a consistently strong positive correlation in both seasons, with higher values in autumn (average around 0.90) compared to spring (average r around 0.84). These correlations are also always statistically significant, with a *p*-value consistently below 0.02. The relatively low standard deviation values (ranging from 0.02 to 0.05) suggest that the correlation remains stable across different measurements, reinforcing the reliability of this trend. The maximum values of r reaching up to 0.95 in autumn and 0.89 in spring indicate periods where the relationship between CO_2_ and RH is extremely strong, whereas the minimum values (down to 0.76 in spring) suggest occasional deviations, likely influenced by specific ventilation patterns or external environmental factors, mainly due to the opening of the windows.

By analyzing the overall results, there is a positive correlation between CO_2_ and RH, as also demonostred for poorly ventilated indoor environments in [20,21]. Specifically, Lazovic et al. [20] found a very strong correlation (greater than 0.80) in aschool with the lowest ventilation rate, due to only manual ventilation. Additionally, the found correlation is greater than the 0.60 found in the study proposed in [30], where a similar case study has been conducted focusing on only natural ventilation. This relationship arises because both parameters share common influencing factors. Human respiration is a significant source of both CO_2_ and water vapor, contributing simultaneously to increased concentrations of both variables in enclosed spaces. When ventilation is insufficient, these emissions accumulate, leading to a concurrent rise in CO_2_ and RH levels. Research has shown that in such conditions, elevated CO_2_ levels are often associated with higher RH due to the shared impact of limited air exchange [31]. This correlation underscores the importance of ventilation as a key factor in maintaining indoor air quality.

From a health and comfort perspective, the seasonal increase in both CO_2_ and RH levels has important implications. Elevated CO_2_ concentrations can lead to reduced cognitive performance, fatigue, and discomfort, particularly when exceeding 1000 ppm [7,8,32]. Similarly, higher RH levels can create favorable conditions for mold growth and dust mites, which can negatively impact indoor air quality and exacerbate respiratory conditions [33].

Therefore, the findings underscore the critical need for effective ventilation strategies and humidity control during autumn to maintain healthy and comfortable indoor environments. In fact, as also suggested by Norbäck et al. [21], the potential decline in learning capacity due to health issues triggered by poor indoor air quality in classrooms requires greater attention.

### 3.2. Thermal Comfort Analysis

**Figure 5:**
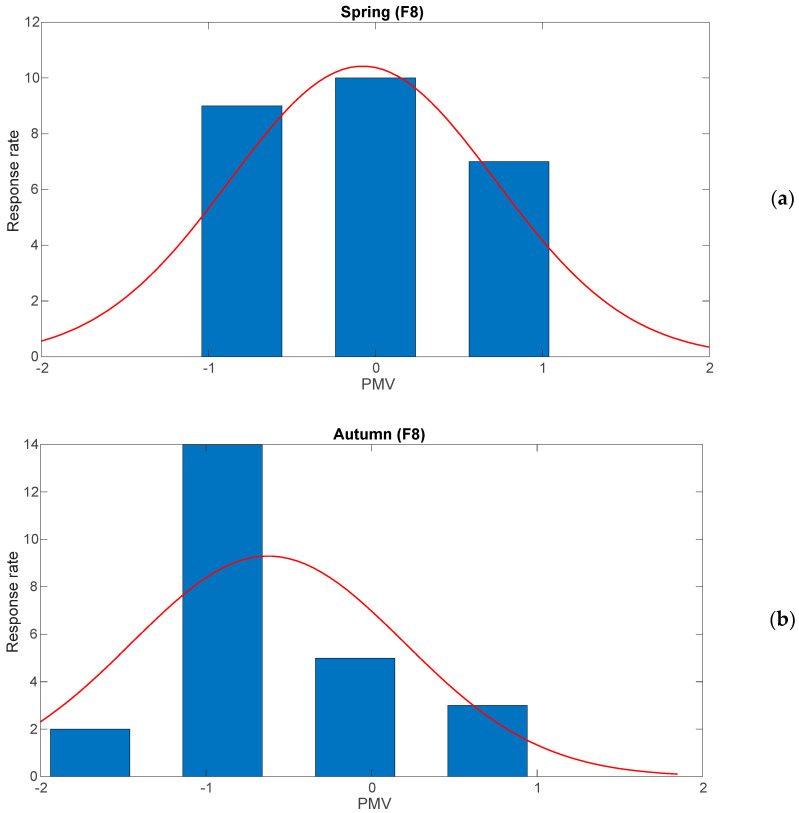
Example of PMV in F8 during spring (**a**) and autumn (**b**).

Considering the data acquired by the BABUC, it was possible to conduct the thermal comfort analysis through the computation of PMV and PPD. Figure 5a,b show an example of the PMV curves obtained in spring and autumn in F8. Generally, in spring, the PMV values exhibit a relatively balanced distribution around neutrality, with the majority of responses concentrated between −1 and +1. The highest frequency is observed at PMV = 0, indicating that most students experienced neutral thermal conditions. This suggests that indoor environmental parameters, such as temperature, humidity, and ventilation, were relatively well regulated, maintaining comfort within acceptable limits. The overall distribution in spring appears symmetric, with no extreme deviations toward discomfort levels. In contrast, in autumn, the PMV distribution shifts, with a notable increase in responses at PMV = −1 and a reduction at PMV = 0 and +1. This shift implies that a larger proportion of occupants perceived the classroom as slightly cool, potentially indicating lower indoor temperatures or differences in thermal insulation due to seasonal variations. The asymmetry in the autumn distribution suggests that a subset of students may have experienced mild thermal discomfort, likely due to inadequate heating or ventilation adjustments to compensate for external temperature changes.

Such results align with previous research indicating that classroom environments often face challenges in maintaining optimal thermal comfort due to varying metabolic rates, clothing insulation, and localized air distribution [34,35]. Overall, these results imply that students perceive a slight thermal discomfort in the classroom, though it is not significant enough to adversely affect the learning environment. However, the persistence of dissatisfaction under certain conditions emphasizes the need for further optimization of HVAC systems, such as improved ventilation, individualized control systems, or adaptive comfort measures, to enhance thermal comfort and support cognitive performance [36].

By considering both CO_2_ concentration analysis and thermal comfort results, the comparative analysis between the two seasons highlights the importance of adaptive thermal management strategies in educational environments. While conditions in spring seem to align more closely with optimal CO_2_ concentration and thermal comfort, autumn exhibits a trend toward cooler perceptions. Thus, the need is evident for targeted interventions, such as enhanced ventilation and humidity control, to mitigate indoor air quality challenges in cooler seasons.

### 3.3. Policy Implications and Recommendations

The findings of this study underscore the need for universities to adopt targeted strategies for maintaining optimal indoor environmental quality, particularly in response to seasonal variations in CO_2_ concentration and thermal comfort. In fact, given the observed increase in CO_2_ levels and relative humidity during autumn, institutions should prioritize enhanced ventilation strategies, such as a combination of natural and mechanical ventilation systems, to maintain air exchange rates within recommended thresholds. Implementing automated ventilation controls that adjust airflow based on real-time CO_2_ levels could help mitigate periods of poor air quality, especially in high-occupancy classrooms.

Additionally, real-time air quality monitoring systems should be integrated into classroom management practices. Displaying CO_2_ concentration levels in lecture halls through visual indicators can promote timely interventions, such as opening windows or adjusting mechanical ventilation settings. Universities should also establish policies that encourage regular ventilation breaks during lessons, particularly in colder months when natural ventilation is often reduced. From a long-term perspective, educational institutions should consider investing in advanced HVAC systems with demand-controlled ventilation (DCV) technology, which automatically adjusts airflow based on occupancy and air quality conditions. Future campus renovations and new building designs should incorporate these principles to ensure a healthier and more conducive learning environment. By implementing these recommendations, universities can significantly enhance indoor environmental quality, contributing to improved student concentration, well-being, and overall academic performance.

### 3.4. Limitations

While this study provides valuable insights into CO_2_ concentration and thermal comfort in university classrooms, some limitations should be acknowledged. First, the analysis focused exclusively on CO_2_ as an indicator of ventilation efficiency, without considering other air quality parameters such as particulate matter (PM), volatile organic compounds (VOCs), or other indoor pollutants that could further influence the learning environment. Additionally, this study was conducted within a single university in Italy, meaning the findings may not be fully generalizable to institutions in different climatic regions, building designs, or ventilation configurations. Future studies should explore similar analyses in a wider range of educational settings to enhance the applicability of the results.

Another limitation is the lack of direct cognitive performance assessments. While high CO_2_ levels have been linked to impaired cognitive abilities in previous literature, this study did not include objective measurements such as student test performance, concentration levels, or physiological indicators like heart rate variability. Incorporating such data in future research could provide stronger evidence of the impact of CO_2_ and thermal comfort on student well-being and academic performance. Lastly, the thermal comfort assessment was based on subjective self-reported data, which, despite being a widely used method, is inherently influenced by individual perception differences. A more comprehensive evaluation that integrates objective thermal indicators, such as skin temperature or metabolic rate, could offer a more accurate representation of students’ thermal comfort. Despite these limitations, this study provides a strong foundation for understanding seasonal variations in CO_2_ concentration and thermal comfort, highlighting the need for improved ventilation strategies in university classrooms.

## 4. Conclusions

This study provides valuable insights into the seasonal variations of CO_2_ concentration and thermal comfort in university classrooms, emphasizing the importance setoff setting appropriate ventilation strategies for indoor environmental quality. The results indicate that during autumn, reduced ventilation leads to a significant increase in CO_2_ levels, with peak values exceeding recommended thresholds. This, combined with higher relative humidity, suggests inadequate air exchange. In contrast, spring conditions allow for maintaining CO_2_ concentrations within safer limits and contributing to a more comfortable learning environment. A strong positive correlation between CO_2_ concentration and relative humidity was observed, reinforcing the role of ventilation in regulating indoor conditions. The findings demonstrate that ineffective ventilation not only leads to air stagnation but also increases humidity levels, which can further impact occupant comfort. The analysis of thermal comfort through the Predicted Mean Vote (PMV) index highlighted seasonal differences in perceived comfort, with students reporting slightly cooler sensations in autumn, likely due to temperature variations and heating system efficiency.

These findings underscore the need for targeted ventilation strategies that adapt to seasonal changes. Educational institutions should implement optimized ventilation protocols, combining natural and mechanical ventilation to maintain CO_2_ levels within recommended thresholds. Real-time air quality monitoring systems could provide valuable data to support dynamic ventilation adjustments, improving indoor conditions and enhancing student performance. Future research should further investigate the impact of CO_2_ exposure on cognitive function through direct performance assessments and physiological measurements. Additionally, expanding this study to different climatic regions and educational settings would provide a more comprehensive understanding of ventilation efficiency and its influence on indoor environmental quality. Exploring the integration of automated air exchange systems and advanced climate control technologies could lead to significant improvements in university classroom environments, fostering better health and learning outcomes for students.

## Figures and Tables

**Table 1:** Metrological characteristics of Fybra sensors.

Variable	Measurement Range	Accuracy	Repeatability	Response Time
CO_2_	0–40,000 ppm	±(40 ppm + 5% reading error)	±10 ppm	60 s
RH	0–100%RH	±9%RH	±0.4%RH	90 s
T	−10–60 °C	±1.5 °C	±0.4 °C	120 s

**Table 2:** Metrological characteristics of the BABUC system.

Sensor	Operative Range	Accuracy
Hot-wire anemometer	0–50 m/s	±0.05 m/s
Globe thermometer	−50–100 °C	±0.17 °C
Psychrometer	−50–150 °C	±0.13 °C
	40–100%RH	±2%RH

**Table 3:** Students’ occupancy during case study and demographic information.

	Season	Avg. Occupancy	Max Occupancy	Gender (%Male/%Female)	Avg. Height (m)	Avg. Body Mass (kg)	Avg Age (y.o.)
F8	Spring	27	30	70/30	174 ± 7	71 ± 12	22 ± 3
	Autumn	26	30	70/30	174 ± 7	71 ± 12	22 ± 2
F9	Spring	23	27	75/25	173 ± 7	72 ± 10	22 ± 2
	Autumn	23	28	77/23	172 ± 6	73 ± 10	22 ± 2
B1	Spring	29	30	65/35	175 ± 7	68 ± 11	25 ± 1
	Autumn	28	30	64/36	176 ± 8	71 ± 11	25 ± 2

**Table 4:** Seven-point thermal sensation scale.

PMV	Thermal Sensation
+3	Very warm
+2	Warm
+1	Slightly warm
0	Neutral
−1	Slightly cold
−2	Cold
−3	Very cold

**Table 5:** Mean (SD) of the mean, maximum and minimum value of temperature (T), relative humidity (RH) and CO_2_ concentration for each room, considering the whole day, divided for the two seasons. * indicates statistical differences between seasons.

		F8			F9			B1		
		*Mean*	*Max*	*Min*	*Mean*	*Max*	*Min*	*Mean*	*Max*	*Min*
**SPRING**	**T** **(°C)**	23.5 * (0.8)	24.6 (1.3)	22.6 (1.1)	23.9 * (0.9)	27.5 (4.0)	22.4 (1.1)	24.5 * (0.7)	25.6 (0.9)	23.4 (0.6)
	**RH** **(%)**	36.5 * (2.1)	39.4 (2.7)	32.7 (3.7)	32.7 * (1.8)	37.2 (2.6)	27.4 (3.7)	34.1 * (3.0)	38.1 (3.8)	30.3 (4.3)
	**CO_2_ (ppm)**	492.0 * (62.4)	729.8 (280.1)	422.71 (33.8)	503.0 * (36.3)	953.4 (286.7)	419.2 (12.6)	547.4 * (84.6)	934.6 (200.1)	425.5 (32.0)
**AUTUMN**	**T** **(°C)**	21.0 * (1.1)	24.0 (1.0)	19.1 (1.5)	21.5 * (0.9)	23.5 (0.5)	20.4 (1.0)	22.9 * (1.3)	24.5 (2.1)	20.9 (2.5)
	**RH** **(%)**	51.6 * (5.3)	55.6 (6.5)	47.1 (6.6)	50.0 * (4.0)	51.8 (4.8)	47.0 (4.4)	49.4 * (3.9)	54.2 (4.2)	46.0 (4.7)
	**CO_2_ (ppm)**	1034.0 * (153.9)	2324.2 (524.0)	457.2 (51.7)	664.4 * (92.1)	1419.6 (224.5)	475.8 (84.5)	702.8 * (92.5)	1399.4 (91.9)	457.0 (22.1)

**Table 6:** Correlation values between RH and CO_2_ concentration.

		r-Value			
	Season	Mean	STD	Min	Max
F8	Spring	0.84	0.03	0.78	0.89
	Autumn	0.90	0.04	0.85	0.95
F9	Spring	0.85	0.02	0.80	0.88
	Autumn	0.91	0.03	0.87	0.94
B1	Spring	0.83	0.04	0.76	0.88
	Autumn	0.89	0.05	0.83	0.93

## Data Availability

The dataset presented in this article are not readily available because the data are part of an ongoing European project. Requests to access the datasets should be directed to juri.taborri@unitus.it.
